# Studies on Chemical Composition and Antioxidant Activity of *Rudbeckia triloba*

**DOI:** 10.1155/2017/3407312

**Published:** 2017-11-27

**Authors:** Zenovia Moldovan, Mihaela Buleandră, Eliza Oprea, Zamfirica Mînea

**Affiliations:** ^1^Department of Analytical Chemistry, Faculty of Chemistry, University of Bucharest, 90–92 Panduri Av., 050663 Bucharest, Romania; ^2^Department of Organic Chemistry, Biochemistry and Catalysis, Faculty of Chemistry, University of Bucharest, 90–92 Panduri Av., 050663 Bucharest, Romania

## Abstract

The paper describes the physicochemical studies made on the decorative plant, *Rudbeckia triloba* (Asteraceae). For this purpose, essential oil, infusion, decoction, and hydroalcoholic macerate obtained from different aerial parts of *Rudbeckia triloba* were analyzed. The main phytochemical constituents identified by GC-MS analysis were found to be α-pinene (in dried leaves (46.0%) and flowers (40.1%)) and β-phellandrene (in essential oil of dried inflorescences (26.09%)). The Folin–Ciocalteu and quercetin assays revealed different values of total phenolic and flavonoid contents of petals, leaves, and seeds as a function of the solvent used and extraction procedure. The hydroalcoholic macerate of petals was found to present the maximum phenolic and flavonoid contents (130.29 ± 5.58 mg gallic acid equivalent/g dry vegetable material and 30.72 ± 1.35 mg quercetin equivalent/g dry vegetable material, resp.) and also exhibits the lower value of EC50 (0.32% (v/v)), obtained by applying the DPPH· assay. Comparing the extraction methods applied, the maceration was found to be the most effective for phenolic compounds, most likely due to the solvent (70% ethanol). The use of water-alcohol mixture leads to an improvement of the extraction yield of phenolic compounds (including those with higher molecular weights) than by using water as extractive solvent, in the case of infusions and decocts.

## 1. Introduction

For centuries, herb plants are used as a source of medicine. World Health Organization (WHO) reports that the world's population resort to folk medicine for their primary healthcare needs [1]. In this context, natural products, such as plant extracts, either as pure compounds or as standardized extracts, provide unlimited opportunities for new drug discoveries [2]. Also, vegetable materials are used from ancient time as source of flavoring, beverages, fragrances, and cosmetics products. This is because the plant materials have unique properties to synthesize biologically active compounds resulted as metabolites from their so-called secondary metabolism [3]. Between the four major classes of bioactive secondary metabolites (terpenes, phenolics, glycosides, and alkaloids), the phenolic compounds (derived from secondary metabolism of the aromatic amino acids) have antioxidant, anticarcinogenic, and antimutagenic activities, being of particular importance for human health [4]. They are also known to decrease cardiovascular risks [5]. Epidemiological studies demonstrated the ability of plant polyphenols to offer protection against development of cancers, cardiovascular diseases, diabetes, osteoporosis, and neurodegenerative diseases [6]. The phenolic groups in polyphenols accept an electron to form relatively stable phenoxyl radicals, disrupting chain oxidation reactions in cellular components [7]. Phenolics represent a large variety of compounds such as simple phenols, phenolic acids, coumarins, flavonoids, stilbenes, hydrolysable and condensed tannins, lignans, and lignins [8]. It should be noted that flavonoids represent one of the most important natural phenols. Another important class of compounds occurring in plant materials is represented by terpenoids, which play an essential role in development of living systems. Most of them are volatile substances and are generally present in the essential oil obtained by extractive methods.

Not just perennial but also cultured plants are studied in terms of their pharmacological properties. Between cultured plants, the decorative species of *Rudbeckia* are present in particular or public gardens. Native to North America, *Rudbeckia* is a flowering plant of the family Asteraceae, found in 25 different perennial, annual, or biennial species. The most common varieties of this plant are *Rudbeckia fulgida*, *Rudbeckia hirta*, *Rudbeckia laciniata*, or *Rudbeckia triloba*. This last one was reported in Austria in the 1970s, while in Romania, it has been encountered for the first time in Maramures and Neamt counties [9].

Few studies on biochemical properties of *Rudbeckia* varieties are reported [10]. Thus, leaf essential oils from three herbaceous members of the Asteraceae family, *Rudbeckia fulgida*, *Rudbeckia hirta*, and *Symphyotrichum novae-angliae*, were obtained by hydrodistillation and analysed by gas chromatography-mass spectrometry. The main components in the essential oil of *Rudbeckia fulgida* were found to be the sesquiterpene hydrocarbons (germacrene D (30.1%) and δ-cadinene (17.8%)); similar composition was reported in the case of leaf oil of *Rudbeckia hirta*, with 23.6% germacrene D and 16.2% δ-cadinene. The leaf oils of these extracts were found to be inactive for antimicrobial activity [11]. Another study reports the chemical composition, minerals, and antioxidative effect of *Rudbeckia laciniata* [12]. General component of *Rudbeckia laciniata* is crude protein. The highest content of minerals is potassium, followed by calcium and magnesium, suggesting that *Rudbeckia laciniata* was alkaline material. The content of total phenolic and flavonoid compounds and DPPH· radical scavenging activity of *Rudbeckia laciniata* were also reported. The chemical profile of *Rudbeckia hirta* was established by thin-layer chromatography (TLC) technique and by means of the total content of polyphenols [13]. As about the therapeutic actions of the species of the genus *Rudbeckia*, they are relatively varied. The active compounds or extracts of these plants exhibit anti-inflammatory effects (*Rudbeckia hirta*) [14, 15], antimycobacterial (*Rudbeckia subtomentosa*) [16], antileukemic (*Rudbeckia mollis*) [17], antitussive (*Rudbeckia fulgida*) [18], antioxidant, antibacterial, antiviral, antifungal, and cytotoxic activities (*Rudbeckia laciniata*) [19].

To the best of our knowledge, no study has reported in literature on the chemical composition and antioxidant activity of *Rudbeckia triloba*. In this paper, different aerial parts of the *Rudbeckia triloba* were used to obtain essential oil, infusion, decoct, and hydroalcoholic macerate. The solid plant materials (dried leaves and inflorescences), the essential oil of dried inflorescences, and the obtained aqueous or hydroalcoholic extracts from dried petals, leaves, and seeds were characterized by chromatographic or/and spectrophotometric methods.

## 2. Experimental

### 2.1. Reagents

All chemicals used were of analytical grade. Folin–Ciocalteu's reagent (lithium sulphate 12.2%, disodium wolframate dihydrate 2%, hydrochloric acid 9.5%, phosphoric acid 6.9%, bromine 0.1%, water 67%, disodium molybdate dihydrate 2%, and sodium thiosulphate 0.3%); quercetin, ≥95% (2-(3,4-dihydroxyphenyl)-3,5,7-trihydroxy-4*H*-chromen-4-one); gallic acid, >99% (3,4,5-trihydroxybenzoic acid); and DPPH· (2,2-diphenyl-1-picrylhydrazyl) were purchased from Sigma-Aldrich (Steinheim, Germany). Aluminum chloride anhydrous, sodium carbonate, and potassium acetate were purchased from Merck (Germany). Ethanol 96% p.a. was obtained from Chemical Company (Romania).

### 2.2. Collection of the Plant Material

*Rudbeckia triloba* was collected in August 2016 from particular gardens placed in a limitrophe rural area of Bucharest, Romania (Islaz village, Branesti commune, Ilfov county, latitude: 44.46117°; longitude: 26.38724°; altitude, 70 m; Bucharest distance, 6.92 km). The plant was authenticated at the Bucharest Botanical Garden, where the voucher specimen was deposited (406406/30.03.2017). After collection, the plant material was separated into its principal aerial components: inflorescences, petals, leaves, and seeds which were washed thoroughly with water to remove dust. Then, the vegetable materials were let aside to dry at room temperature. The dried components of the plant were cut into small pieces and kept in paper bags until further experiments.

### 2.3. Preparation of Plant Extracts

Infusion, decoction, and maceration were applied on three aerial parts of the plant, namely, petals, seeds, and leaves. Before infusion and decoction, the weighed amount of dry vegetable material (1 g) was soaked with a small volume of cold bidistilled water and left aside for 10 min. This preliminary procedure allows the plant cells to expand and thus to release their active ingredients when preparing the infusion or decoction. The infusion was prepared by adding 50 mL of bidistilled boiled water to the wet vegetable material. The mixture was covered with a watch glass, left to stand for 15 minutes. The decoction was made by boiling the wet plant material with 50 mL of water for 10 min. After that, the mixture was left aside for 15 min. The hydroalcoholic extract was prepared by macerating 0.5 g of the vegetable material in 50 mL of 70% ethanol for 7 days, in dark, at ambient temperature. Then, each plant extract was filtered through a filter paper. The filtrate was quantitatively transferred to a 100 mL volumetric flask and brought to the mark with bidistilled water (in the case of infusion and decoct) and with 70% ethanol (in the case of macerate), to obtain the diluted extracts. Parts of the obtained filtrate extracts were stored at −45°C for further analyses. The essential oil was isolated from dried inflorescences, by hydrodistillation in a Clevenger-type apparatus, for 4 h, using a standard procedure [20]. The essential oil was dried on anhydrous Na_2_SO_4_ and stored at +4°C. The quantity of essential oil from plant was expressed in percentage (v/w), as an average of the values obtained from five extractions.

### 2.4. Preparation of Analytical Reagent Solutions

A 500 μg/mL stock solution of gallic acid (GA) was prepared by dissolving 0.0125 g GA in distilled water, brought to 25 mL in a volumetric flask. A working solution of 50 μg GA/mL was obtained by dilution from the stock solution. A stock quercetin (Q) solution of 1 mg/mL was prepared by dissolving 0.0250 g Q in 96% ethanol and brought to 25 mL in a volumetric flask. A working solution of 100 μg Q/mL was obtained from the stock solution, by dilution with 96% ethanol. A 0.05% ethanolic solution of DPPH· was prepared just before use.

### 2.5. GC-MS Analysis

The GC-MS analyses were carried out with a Thermo Electron system (Focus GC chromatograph coupled with a Polaris Q ion trap mass detector). A DB-5MS capillary column (25 m × 0.25 mm; 0.25 μm of film thickness) was used with helium as carrier gas (1 mL/min). GC oven was programmed at an initial temperature of 60°C for 3 min. The temperature was increased up to 200°C at the rate of 10°C/min, kept constant for 2 min, and then increased up to 240°C at the rate of 12°C/min and held at 240°C for 2 min [21]. Both injector and detector temperatures were 250°C. The electronic impact ionization mode was 70 eV, and the detection was performed in the range of 35–300 amu (full scan mode). The identification of the components was carried out using Xcalibur® software, NIST Mass Spectral Library, and MS literature data. Retention indices (RIs) of the compounds were determined relative to the retention times of *n*-alkanes standard solution for GC (C8–C20). Relative percent of individual components was calculated based on the GC peak areas without the use of correction factors. For GC-MS analysis, the hydrodistilled essential oil was diluted in hexane (1 : 100) and 1 µL was injected. A Triplus HS Autosampler (Thermo Electron) was used for headspace analysis, an amount of 1 g of inflorescences or leaves being introduced in a sample vial (20 mL size) and sealed with silicone rubber septum and aluminium cap. The headspace vial containing the plant material was heated to 80°C for 10 min, and 500 μL of the headspace gas was injected into the column. The GC-MS analysis was performed in the same way as for essential oils.

### 2.6. Determination of Total Phenolic Content (TPC)

All absorbance measurements were made on a UV-Vis spectrometer (Jasco V-530, Jasco, Japan), equipped with quartz cells (1 cm light path length).

The spectrophotometric Folin–Ciocalteu (F–C) assay [22] was used to determine the total polyphenolic content. Aliquots (0.1–0.5 mL) of the working gallic acid solution (50 μg/mL) were put into a series of 5 mL-calibrated flasks. Then, 2.5 mL of 10% the Folin–Ciocalteu reagent was added. The mixtures were incubated in darkness for 5 min. Then, 2 mL of 7.8% sodium carbonate solution was added to each sample, and the total volumes were brought to 5 mL with distilled water. After homogenization, the mixtures were incubated in darkness for 30 min before measuring absorbance at 760 nm against blank solution (obtained without adding gallic acid). A standard calibration curve was prepared in the concentration range of 1–5 μg/mL gallic acid. For determination of the total phenolic content in the extracts, an aliquot of each diluted extract was used instead of gallic acid, and the F–C procedure was applied. The total phenolic content was calculated in terms of gallic acid equivalents per gram of dry vegetable material (mg GAE/g), by applying the regression equation of the calibration curve *A* = 0.0968*C*_GA_ + 0.0197 with *R*^2^ = 0.9998, where *A* is the absorbance and *C*_GA_ is the concentration of gallic acid solution (μg/mL). The spectrophotometric measurements for each sample were made in triplicate.

### 2.7. Determination of Total Flavonoid Content (TFC)

The flavonoid content was determined spectrophotometrically, on the basis of Al(III)-flavonoid complexes formation. Quercetin was used as the standard flavonoid, and the cited procedure was applied with little modification [23]. Aliquots (0.1–0.5 mL) of the working quercetin solution (100 μg/mL) or diluted extract samples and ethanol (up to 2 mL alcoholic mixture) were introduced in a series of 5 mL-calibrated flasks. Subsequently, 0.1 mL of 10% aluminum chloride, 0.1 mL of 1M potassium acetate solutions, and water were added to each sample to make the total volume of 5 mL. The mixtures were homogenized and left aside for 30 min at room temperature. Then, the absorbance was measured at 427 nm against reagent blank (for each sample, a blank mixture was prepared, without adding AlCl_3_). The total flavonoid content of the vegetable materials was calculated in terms of quercetin equivalents per gram of dry vegetable material (mg QE/g), by applying the regression equation of the calibration curve *A* = 0.0652*C*_Q_ + 0.0804 with *R*^2^ = 0.9992, where *C*_Q_ is the concentration of quercetin solution (μg/mL). The spectrophotometric measurements for each sample were made in triplicate.

### 2.8. DPPH· Radical Scavenging Assay

The DPPH· free radical scavenging activity of the obtained herbal extracts was determined according to Shimada et al. with slight modification [24]. Aliquots of filtrate extracts (0.01–0.3 mL) were mixed with ethanol up to 1.9 mL. Then, 0.1 mL of 0.05% ethanolic solution of DPPH· was added to each sample up to a total volume of 2 mL. A standard solution containing 0.1 mL of 0.05% DPPH· and 1.9 mL ethanol was also prepared. The mixtures were homogenized and kept in darkness for 30 min, at room temperature. Then, absorbance measurements were made at 517 nm against reagent blank (for each sample, a blank mixture was prepared without adding DPPH·). The DPPH· solution was freshly prepared and kept in a refrigerator until used. EC50 value was obtained by plotting the absorbance at 517 nm versus concentration of extract solutions. The concentration (%, v/v) of the sample solution that decreases the initial absorbance of DPPH· by 50% represents EC50.

## 3. Results and Discussion

### 3.1. Essential Oil Yield

The average content in the essential oil of *Rudbeckia triloba* inflorescences was 0.300% (mL essential oil/100 g plant). The essential oil was light yellow with a specific odour.

### 3.2. Results of GC-MS Analysis

The GC-MS analysis revealed that the number of volatile components of the hydrodistilled essential oil of inflorescences was different from that of the dry plant material (leaves and inflorescences) undergoing headspace extraction.  Table 1 shows the relative content of volatile compounds from leaves, inflorescences, and essential oil of inflorescences of *Rudbeckia triloba* growing in Romania, expressed as percentage from the total area. Thus, using headspace, twenty-two compounds were identified in leaves accounting for 99.0% of the total identified components, with α-pinene (46.0%), β-phellandrene (24.6%), sabinene (9.6%), and germacrene D (6.1%) as the main components. The flowers of *Rudbeckia triloba* contained thirty-three components (98.6%), with the same principal constituents, but in different percentages: α-pinene (40.1%), germacrene D (24.0%), β-phellandrene (13.9%), and sabinene (9.7%). The total number of identified components of the essential oil of inflorescences was forty-one, representing 95.9% of total areas. The same components were the main volatile compounds of the essential oil, with β-phellandrene (26.09%), germacrene D (21.6%), α-pinene (16.3%), and sabinene (12.0%).

On the basis of the GC-MS analysis, one can conclude that *Rudbeckia triloba* is an important source of α-pinene. This hydrocarbon monoterpene is known to possess anxiolytic and sedative properties [25]. It also acts as a bronchodilator. It was reported that this terpene has the property to reduce the size of cancerous tumors [26].

### 3.3. Total Phenolic and Flavonoid Contents

The major part of the reported studies concluded that polyphenols (flavonoids being the largest family of polyphenolic compounds) are the principal compounds responsible for the antioxidant activity of the tested plant extracts [27].  Table 2 presents the TPC and TFC of the obtained extracts (infusion, decoct, and hydroalcoholic macerate of different aerial parts of *Rudbeckia triloba*).

The maximum TPC and TFC values were obtained by maceration of the vegetable materials followed by decoction and infusion. All extraction procedures showed maximum values of TPC and TFC in petals. A comparison between infusion and decoction shows that decoction is more efficient than infusion in polyphenol extraction. This behavior could be explained as follows. Both infusion and decoction are thermal procedures suitable for extracting heat-stable compounds leading to differences in the total phenolic content of decoct and infusion. The higher concentration of the total phenolic content in decoct than in infusion, obtained by heating the water-vegetable mixture for a long time (15 min), could be explained as follows: breakdown of cellular constituents as well as hydrolysis of tannins takes place. The degradation of complex phenolic tannins as well as the enzymatic or nonenzymatic oxidation process leads to supplementary content of phenolic compounds in decoct. At the same time, during the decoction, the Maillard reaction takes place, producing new phenolic compounds [28].

Also, comparing the three applied procedures of extraction (infusion, decoction, and maceration), maceration seems to be more efficient than infusion and decoction for extracting polyphenols (70% ethanol was chosen for maceration, being the most used solvent for extracting phenolic compounds from plants). This behavior could be caused by possible complex formation of some phenolic compounds, possessing more phenol groups or having higher molecular heights than the phenolic in water. These compounds seem to be soluble in hydroalcoholic extract. Also, the content of more nonphenol compounds, such as terpene, organic acids, sugars, and soluble proteins, in infusion than in macerate could interfere in the TPC assay. It may also be caused by the possible complex formation of some phenolic compounds in the macerate extract that are soluble in ethanol-water mixture. These phenolic compounds may possess more phenol groups or may have higher molecular weights than the phenolics in the water extract [23]. As reported, a water-alcohol mixture used in the extraction procedure leads to an increase in swelling of the plant materials, assuring an intimate contact between the plant matrix and the solvent and consequently the improvement of the extraction yield [29]. In addition, the plant materials contain a large variety of phenolic compounds having different numbers of phenolic groups with different reactivities towards the Folin–Ciocalteu reagent [30].

As about flavonoids, from the data reported in the literature, the major assays for the total flavonoids content determination are generally based of the absorbance of Al(III)-flavonoids compounds [31–34]. The difference between the two mostly applied spectrophotometric methods is the reaction medium: (a) in the presence of NaNO_2_, in alkaline medium and (b) in the presence of CH_3_COOK or CH_3_COONH_4_ (without nitrite). In our work, the spectrophotometric method in the absence of nitrite was used. This last one method can be only used to determine the content of flavonols (quercetin, rutin, kaempferol, and morin) and luteolin (from flavones family) [35]. As about correlation between TPC and TFC, it was found to be significant in the case of infusion and maceration (Table 3). On the basis of literature data, such a correlation indicates that flavonoids represent an important part of the phenols in plant extracts [23]. The results in Table 2 show that infusions and macerates obtained from petals and leaves seem to contain significant percents of flavonoids.

### 3.4. Free Radical Scavenging Activity

As mentioned, polyphenolic and flavonoid compounds are considered to have significant antioxidant properties mainly due to their important content in hydroxyl groups. These substances are able to act as radical scavengers because phenolic hydroxyl groups are good H-donating antioxidants, which scavenge reactive oxygen species and stop the production of new radicals. The stable DPPH· radical is often used to assess the radical scavenging activity of many biological active compounds. DPPH· has the property to accept an electron from an antioxidant. This redox reaction leads to the decrease of the violet color of the DPPH· radical. The total reaction antioxidant-DPPH· leads to a yellow-colored solution. A low EC50 value denotes a high scavenging activity. As observed in Table 4, the extracts obtained from petals present the lowest values of EC50 with a minimum in the case of macerate extract. As about relationship between EC50 and TPC values, generally, negatively linear correlations are reported in literature. But, there is not always a linear correlation between these two analytical parameters. In our study, negative linear correlations were obtained in the case of infusions and macerate extracts (Table 5). Cheynier et al. assumed that besides the radical scavenging, the antioxidant activities could also be attributed to other different mechanisms such as binding of transition metal ion catalysts or decomposition of peroxides [36]. At the same time, the radical scavenging activity of polyphenols depends on their molecular structure, respectively, on the availability of phenolic hydrogen and the possibility of stabilization of the resulting HO and NO radicals via hydrogen donation [37].

## 4. Conclusion

The obtained results on *Rudbeckia triloba* extracts indicate significant antioxidant activity, especially of macerate of petals (resulted by applying Folin–Ciocalteu, total flavonoids, and DPPH· assays). Also, leaves, inflorescences, and the inflorescences' essential oil of *Rudbeckia triloba* were found to be rich in monoterpene hydrocarbons (especially, α-pinene in leaves and inflorescences and β-phellandrene and germacrene D, in inflorescence essential oil). Further studies will complete the characterization of *Rudbeckia triloba*. They will be focused both on the complete analysis of the composition of the aqueous and hydroalcoholic *Rudbeckia triloba* extracts and on their biochemical characterization.

## Figures and Tables

**Table 1:** Compounds identified by GC-MS technique in the *Rudbeckia triloba*.

Number	Compound	RI^a^	Composition^b^ (%)		
			Leaves	Inflorescences	
				Dry material	Essential oil
1	α-Thujene	924	0.2	0.2	—
2	α-Pinene	940	46.0	40.1	16.3
3	Camphene	957	0.2	0.5	0.3
4	Sabinene	978	9.6	9.7	12.0
5	β-Pinene	982	0.8	0.7	—
6	β-Myrcene	991	2.9	1.5	1.7
7	α-Phellandrene	1006	1.1	0.8	1.3
8	3-Carene	1013	0.1	0.2	—
9	α-Terpinene	1018	—	—	0.2
10	*p*-Cymene	1026	0.6	0.2	—
11	β-Phellandrene	1031	24.6	13.9	26.0
12	Z-β-Ocimene	1046	0.3	0.4	0.6
13	Butyl-2-methylbutanoate	1048	0.2	—	—
14	E-β-Ocimene	1055	0.3	0.3	—
15	γ-Terpinene	1059	0.2	0.0	0.4
16	*cis*-Sabinene hydrate	1071	—	0.1	—
17	α-Terpinolene	1088	0.8	—	0.2
18	Linalool	1094	0.3	0.1	—
19	*trans*-*para*-Mentha-2,8-dien-1-ol	1108	—	0.7	—
20	Α-Campholenal	1127	—	0.7	0.3
21	*cis*-*p*-Mentha-2,8-dien-1-ol	1136	—	—	0.1
22	Sabinol	1142	0.2	0.2	0.2
23	*cis*-Verbenol	1147	—	0.3	0.6
24	Lavandulol	1165	—	—	0.2
25	*trans*-2-Caren-4-ol	1172	—	0.1	0.3
26	Terpinen-4-ol	1179	—	—	0.5
27	Cryptone	1190	—	0.6	1.0
28	Verbenone	1216	—	0.2	—
29	*trans*-Carveol	1219	—	—	0.1
30	Butanoic acid, 2-methyl-, hexyl ester	1230	0.6	—	—
31	Cumin aldehyde	1244	—	—	0.4
32	Phellandral	1281	—	—	0.2
33	δ-Elemene	1339	—	—	0.1
34	α-Terpineol acetate	1345	2.5	—	—
35	Cyclosativene	1374	—	—	0.1
36	Α-Copaene	1379	—	—	0.1
37	Β-Maaliene	1388	—	—	0.1
38	Β-Cubebene	1392	—	0.2	0.5
39	β-(E)-Caryophyllene	1427	0.4	1.2	1.7
40	Β-Gurjunene	1435	—	0.3	0.6
41	*cis*-β-Farnesene	1450	—	—	0.3
42	Humulene	1461	—	0.2	0.4
43	Γ-Gurjunene	1464	—	0.2	0.4
44	Γ-Muurolene	1480	—	0.2	0.6
45	Germacrene D	1490	6.1	24.0	21.6
46	Α-Muurolene	1503	—	0.3	0.5
47	Γ-Cadinene	1523	—	0.3	0.9
48	Δ-Cadinene	1526	—	—	0.2
49	Spathulenol	1588	—	—	0.9
50	Caryophyllene oxide	1597	—	0.1	0.7
51	Cedr-8-en-13-ol	1656	—	—	0.2
52	Cadalene	1680	1.0	0.1	—
53	6-Isopropenyl-4,8a-dimethyl-1,2,3,5,6,7,8,8a-octahydro-naphthalen-2-ol	1694	—	—	2.2
54	Hexahydrofarnesyl acetone	1747	—	—	0.9
	Total (%)		99.0	98.6	95.9
	Monoterpene hydrocarbons		87.7	68.6	59.0
	Oxygenated monoterpenes		3.0	2.9	3.9
	Sesquiterpene hydrocarbons		7.5	27.0	28.0
	Oxygenated sesquiterpene		—	0.1	4.9
	Nonterpene ester		0.8	—	—

Notes: ^a^retention index; ^b^composition calculated based on the peak area % of each fraction.

**Table 2:** Total phenolic content (TPC) and total flavonoid content (TFC) of extracts obtained from different aerial parts of *Rudbeckia triloba*.

Vegetable material	TFC (QE, mg/g dry vegetable)			TPC (GAE, mg/g dry vegetable)		
	Infusion	Decoct	Macerate	Infusion	Decoct	Macerate
Petals	8.66 ± 0.52	12.69 ± 0.52	30.72 ± 1.35	40.51 ± 2.11	73.53 ± 3.51	130.29 ± 5.58
Leaves	3.22 ± 0.22	2.37 ± 0.11	21.72 ± 1.22	15.02 ± 0.82	62.06 ± 3.12	93.43 ± 4.85
Seeds	0.64 ± 0.06	2.82 ± 0.23	6.65 ± 0.34	5.51 ± 0.32	70.64 ± 3.25	70.14 ± 3.45

**Table 3:** Correlations between total phenolic content and flavonoids content of extracts obtained from different aerial parts of *Rudbeckia triloba*.

Extract	Correlation (*R*^2^)
Infusion	*y* = 0.2259*x* – 0.4251 (0.9971)
Decoct	*y* = 0.7059*x* – 42.568 (0.5212)
Macerate	*y* = 0.3739*x* – 17.183 (0.9147)

**Table 4:** Total antioxidant capacity of extracts obtained from different aerial parts of *Rudbeckia triloba*.

Vegetable material	EC50 (% (v/v))		
	Infusion	Decoct	Macerate
Petals	0.51 ± 0.03	0.61 ± 0.03	0.32 ± 0.02
Leaves	1.01 ± 0.05	2.52 ± 0.13	0.62 ± 0.03
Seeds	2.22 ± 0.11	3.41 ± 0.17	0.88 ± 0.04

**Table 5:** Correlations between total antioxidant capacity and total phenolic content of extracts obtained from different aerial parts of *Rudbeckia triloba*.

Extract	Correlation (*R*^2^)
Infusion	*y* = −18.132*x* + 42.952 (0.7760)
Decoct	*y* = −1.8133*x* + 72.736 (0.1939)
Macerate	*y* = −86.751*x* + 152.73 (0.9504)
